# The ACLGIM LEAD Program: a Leadership Program for Junior-Mid-Career Faculty

**DOI:** 10.1007/s11606-021-06918-y

**Published:** 2021-06-09

**Authors:** April S. Fitzgerald, Michele Fang, Rita S. Lee, Jillian Gann, Deborah L. Burnet

**Affiliations:** 1 Division of General Internal Medicine, Department of Medicine, Johns Hopkins University School of Medicine, Baltimore, MD USA; 2 Division of General Internal Medicine, Department of Medicine, University of Pennsylvania School of Medicine, Philadelphia, PA USA; 3 Division of General Internal Medicine, University of Colorado, Aurora, CO USA; 4 Society of General Internal Medicine, Alexandria, VA USA; 5 Section of General Internal Medicine, University of Chicago Pritzker School of Medicine, Chicago, IL USA

**Keywords:** leadership, faculty, education, networking, mentoring

## Abstract

**Background:**

Junior to mid-career medical faculty often move into administrative and leadership roles without formal leadership training. Many national leadership training programs target senior rather than junior faculty.

**Aim:**

To address the leadership development needs of junior and mid-career faculty.

**Setting:**

Sessions at annual meetings combined with online learning, independent work, and leadership coaching.

**Participants:**

79 junior-mid-career general internal medicine (GIM) faculty enrolled in five consecutive annual cohorts from 2014 to 2018.

**Program Description:**

LEAD scholars participate in a full-day anchor session followed by selected workshops during the annual meeting. They then participate in monthly online sessions, complete a project, interview a senior leader, and receive leadership coaching from senior GIM faculty.

**Program Evaluation:**

Post-program evaluation indicated the LEAD program was effective in helping participants understand what it means to be a good leader (93%, 37/40), become a more reflective leader (90%, 35/39), and apply principles of leadership to increase effectiveness in their role (88%, 34/39).

**Discussion:**

LEAD provides junior-mid-career medical faculty an opportunity to learn effective leadership skills and build a network.

**Supplementary Information:**

The online version contains supplementary material available at 10.1007/s11606-021-06918-y.

## INTRODUCTION

Effective leadership is an essential component of a healthy work environment, shown to increase resiliency and decrease burnout,^1–4^ yet leadership training is often lacking in the path to becoming a physician.^5^ At academic medical centers, physicians commonly move into positions leading educational, clinical, or research programs as junior to mid-career faculty (instructors-assistant professors), but many highly regarded leadership programs available nationally limit the number of participants from an individual institution and are targeted to senior faculty (associate/full professors).^6^ Research on leadership training programs has found that junior (vs. senior) leaders acquire up to four times greater gain from leadership training, possibly due to senior leaders having more entrenched behaviors.^7,8^ Our program aim was to address the leadership development needs of junior to mid-career faculty.

## SETTING AND PARTICIPANT RECRUITMENT

The LEAD (not an acronym) program combined in-person sessions and workshops at the Association of Chiefs and Leaders of General Internal Medicine (ACLGIM) and the Society of General Internal Medicine (SGIM) annual meetings with online asynchronous learning. Participants were recruited through advertisement at SGIM regional and national meetings, posting on the SGIM/ACLGIM websites, and notices to members. Targeted learners were emerging leaders 5–8 years out of training though the program was open to faculty at all ranks, including more senior members due to variance in institution rankings and available training. LEAD scholars were selected through a competitive application process. Applicants submitted a curriculum vitae, personal statement, leadership role(s), and plans for a leadership project. They also obtained a letter of support from their institutional leadership assuring support, time, and resources for participation.

## PROGRAM DESCRIPTION

In 2012, ACLGIM members recognized the need for leadership development for junior faculty and conducted a needs assessment looking at academic faculty leadership strength/weaknesses core competencies (champion change, lead courageously, coach, and develop). These needs were also supported by an SGIM membership survey. In 2013, ACLGIM convened a working group charged with developing the LEAD program.

### Program and Curriculum Development

Two ACLGIM faculty members and one staff member were core to the initial working group formed by the ACLGIM Executive Committee and brought expertise in education, leadership curriculum development, program implementation, faculty development, diversity and inclusion, and mentoring. The resulting Program Development Plan included an overview, mission statement, strength-weakness-opportunity-threat (SWOT) analysis, stakeholder assessment, start-up cost, break-even analysis, staffing requirements, and plan for management, evaluation, and continuous improvement.

We developed the curriculum using Kern’s^9^ six-step approach to curriculum design and anchored in Knowles’^10^ Learning Theory of Andragogy as a framework of learning principles: adult learners need to know the reason for learning something new; they are autonomous and self-directing; they use their prior experience and mental models to provide the basis for learning; adults are most interested in learning subjects with immediate relevance to their work or personal lives; adult learning is problem-centered rather than content-oriented; and adults respond better to internal, rather than external, motivators.

### Program Structure

The LEAD program has four pillars: synchronous sessions, asynchronous sessions, independent work by the LEAD scholar, and coaching by a general internal medicine (GIM) leader. The foundation and start of LEAD is the ACLGIM Leon Hess Management Training and Leadership Institute (Hess Institute) which takes place each spring one day prior to SGIM’s annual meeting. It is a full day, with approximately 100 division chiefs and national GIM leaders in attendance. Most LEAD scholars meet with their assigned GIM coaches on this first day. A breakout session for LEAD at the end of the day allows scholars to receive orientation materials on how to complete each aspect of the program and includes time management training. They also begin group team building. Scholars attend the annual ACLGIM dinner that evening for additional networking opportunities and cohort bonding.

At the SGIM annual meeting of approximately 2000 attendees, the current LEAD cohort and LEAD alumni meet over breakfast to share leadership experiences and challenges. Three 60-min LEAD workshops are offered for LEAD participants, alumni, and any interested SGIM members. Leadership speakers focusing on soft (interpersonal) leadership skills are invited from business schools and leadership institutes in the vicinity of the annual meeting (Appendix A in the Supplementary information). LEAD scholars are expected to attend 2 of the 3 LEAD-sponsored workshops.

Following these initial sessions, LEAD scholars begin the remote asynchronous portion of the program with a monthly online discussion forum which is also vital for cohort bonding. The anchor reading, *HBR’s 10 Must Reads on Leadership*,^11^ is provided to each participant. The discussion forum is secure, accessible only to the LEAD faculty, cohort, and an SGIM staff member. LEAD faculty open the forum each month with a prompt based on the reading, and they guide the discussion while scholars respond to the prompt and reflect on how readings and topics relate to their challenges and experience as emerging leaders. Participants are encouraged to respond to at least two other cohort member postings during the month. The asynchronous nature allows scholars’ flexibility to contribute when their schedules allow. Participation is tracked monthly by faculty.

In addition, each scholar completes a leadership project (identified at the time of application). The scope varies by individual from a personal development goal such as improving their ability to collaborate with others, to an institutional project such as standing up a new service line (Appendix B in the Supplementary information). Flexible parameters allow scholars to be self-directed but require self-motivation, consistent with principles of adult learning. The scholar works on their project throughout the year. LEAD scholars also interview at least one senior leader at their home institution with whom they would not otherwise interact. This component of the program is intended to help the scholar gain further practical insights into leadership while expanding their network at their home institution. LEAD scholars are encouraged to share progress and results with their online LEAD community, as well as their LEAD coach.

LEAD participants are matched with a GIM leadership coach based on mutual interests. Coaches are chosen from among national senior leaders in academic GIM and interact with their scholars to guide and support them in goal setting, projects, leadership reflection, and addressing individual challenges. Coaches are given guidelines for interactions and expectations to hold a monthly 30-min session but do not undergo formal training. Staff send coach reminders, and LEAD faculty check-in with scholars to ensure coach fit and meeting frequency.

LEAD scholars are invited to the ACLGIM Winter Summit each December but are not required to attend. The Summit is an annual 2-day in-person meeting that focuses on timely leadership topics, e.g., policy, equity, burnout. It is a valuable opportunity for scholars to nationally network and meet with coaches. The LEAD program concludes before the occurrence of the next annual ACLGIM/SGIM meeting, so attendance is not required. However, scholars are invited to join the annual ACLGIM dinner to receive formal recognition for completing the program and encouraged to participate in the next LEAD breakfast networking session.

The cost of LEAD is $750 for ACLGIM/SGIM members, $1000/non-members. Participants also attend the ACLGIM Hess Institute and the SGIM annual meeting, which have associated registration and travel costs.

### Participant Description

A total of 79 academic general internists from 53 institutions participated in five annual cohorts (size ranging from 10 to 21) from 2014 to 2018. The participants were 71% (56/79) females. For racial/ethnicity, 9% (7/79) identified as underrepresented in medicine; 24% (19/79) Asian/Asian-Indian/Asian-American (a population underrepresented in medical leadership); 53% Caucasian/White; and 13% other/unspecified. Eighty-two percent (65/79) were instructor/assistant professor or adjunct/no rank; 18% (14/79) were associate/full professor (Table 1).

**Table 1:** LEAD Participants’ Demographics Compared to SGIM and 2018 US Faculty Data

	2014–2018 LEAD Cohort Data	2020* SGIM Member Data	2018† National Faculty Data
Gender, *N* (%)			
Female	56 (71%)	1391 (48%)	45,240 (38%)
Male	23 (29%)	1101 (38%)	72,883 (62%)
Other/prefer not to answer	--	415 (14%)	--
Race/ethnicity, *N* (%)			
African American	4 (5%)	132 (4%)	7583 (6%)
Hispanic/Latino	3 (4%)	107 (4%)	6901 (6%)
Asian/Asian-Indian/Asian-American	20 (25%)	437 (15%)	27,787 (24%)
Caucasian/White	42 (53%)	1510 (52%)	52,210 (44%)
Other /prefer not to answer	10 (13%)	253 (7%)	23,642 (20%)
Current Medical School faculty appointment, *N* (%)			
Instructor	4 (5%)		
Assistant professor	50 (63%)		
Associate professor	13 (16%)		
Full professor	1 (1%)		
Adjunct/none	11 (14%)		
Faculty career path, *N* (%) ∑≠ 100%			
Clinician-educator	61 (77%)		
Clinician-investigator/hybrid^‡^	11 (14%)		
Hospitalist^§^	16 (20%)		
SGIM regions^‖^, *N* (%)			
California-Hawaii	9 (11%)		
Mid-Atlantic	23 (29%)		
Midwest	19 (24%)		
Mountain West	4 (5%)		
New England	10 (13%)		
Northwest	3 (4%)		
Southern	10 (13%)		
International	1 (1%)		

*SGIM member data is a single snapshot in time from November 2020

†https://www.aamc.org/data-reports/workforce/data/table-12-practice-specialty-females-race/ethnicity-2018

‡Hybrid faculty reported both clinician-educator and clinician-investigator roles

§ Hospitalists could include both Clinician-Educator and Clinician-Investigator roles

‖ Information on SGIM regions is located at https://www.sgim.org/communities/regions

## PROGRAM EVALUATION

The LEAD program was evaluated using Kirkpatrick’s Pyramid of Program Evaluation.^12^ Mid-program evaluation was performed for continuous program improvement. A five-cohort post-program alumni survey (Appendix C in the Supplementary information) was sent to all LEAD alumni in September 2019 for program evaluation (RR 51%, 40/79). Respondents indicated the program was most effective at helping participants understand what it means to be a good leader (93%, 37/40), become more reflective as a leader (90%, 35/39), and apply principles of leadership to increase effectiveness in their role (88%, 34/39) (Table 2, Appendix D in the Supplementary information).

**Table 2:**
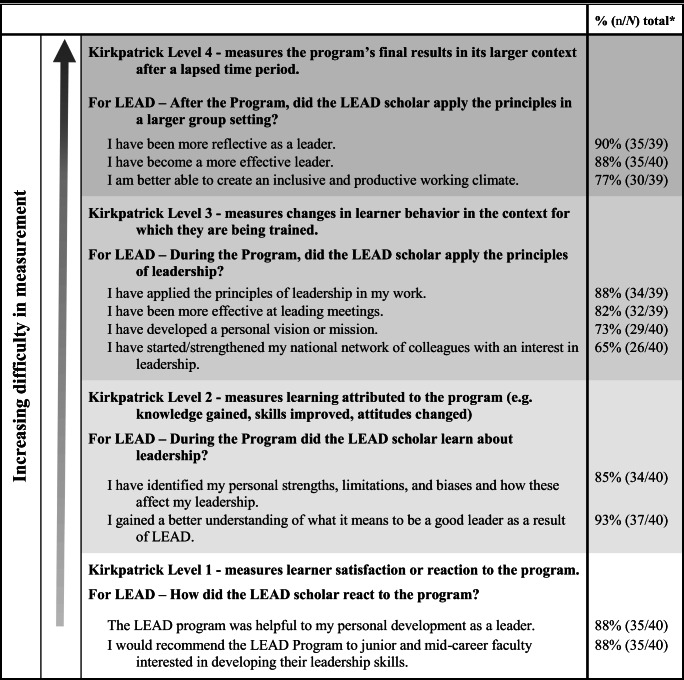
Program Evaluation of LEAD Program by Kirkpatrick Level

*Reported ratings of agree or strongly agree on a 4-point Likert scale (strongly disagree, disagree, agree, strongly agree)

## DISCUSSION

ACLGIM LEAD is an innovative program—grounded in adult learning theory, targeting diverse learners, flexible in its structure, and with a low program cost.

Networking is a key feature of LEAD for many participants. It occurs between cohort members, participant-coach pairs, and among the broader cohort to alumni and GIM leader group. These connections can benefit participants as they continue in academic medicine. Research on networking shows its importance for both job retention and individual performance.^13^ Diverse networks create more value^14^ and lead to better cultural understanding among the members.^15^ An interdisciplinary or interprofessional leadership program might further realize these benefits.

There have been several challenges surrounding the LEAD program. The nature of educating working professionals is one. LEAD’s adult learners face a delicate balance with multiple demands on their time. Personal growth and learning compete with clinical, academic, family, and social responsibilities. LEAD faculty encourage each scholar’s growth with compassion for individual circumstances while helping learners understand that the program operates under a collaborative social constructivist model^16^ where each individual has a responsibility to the community as a whole, e.g., to be accountable, authentic, and collaborative.

Participant demographics demonstrated a high female predominance with racial and ethnic composition mirroring SGIM membership, reflective of national faculty. LEAD is exploring ways to further expand racial and ethnic diversity aligned with SGIM’s effort to improve equity and inclusion.

Senior GIM leaders have been generous with their time as coaches. Practical barriers include both hesitancy on the part of LEAD scholars to contact their coaches and coachs’ obstacles in making contact. Such issues are common challenges in the mentoring environment.^17^ The responsibility for contact between coach and scholar is mutual. Coaches are unpaid volunteers; expressing appreciation to coaches for their willingness to serve the junior faculty is paramount. LEAD faculty publicly recognize coaches whenever possible.

Continuous program monitoring has suggested future improvements including a new learning platform, piloting synchronous online sessions, and enhancing the alumni network.

LEAD program’s goal is to provide structure for leadership training to empower emerging leaders. A meta-analysis looking at leadership training designs suggests that leadership development programs geared toward achieving Kirkpatrick level 4 results should be composed of the following: multiple delivery methods, on-site component with required mandatory attendance, multiple sessions, a longitudinal program (all of which increase effectiveness), and soft skills (intrapersonal, interpersonal, and leadership skills).^8^ LEAD conforms to these evidence-based teaching guidelines.

The program evaluation was limited in reliance on self-reports. Comparison with data from LEAD alumni work environment to corroborate behavior would strengthen evidence for impact. We did not systematically track independent work completion, which is a limitation.

LEAD alumni have continued to progress in their careers after completing their LEAD certificate, moving into more advanced leadership roles within their institutions and professional organizations (Appendix E in the Supplementary information). However, attributing causality to LEAD participation or measuring LEAD by alumni progression might be a misstep. Though a formal leadership title affords positional power, it does not measure ability.

A leader is the individual who knows when to show (or not) support for those above, care for those below, and reach out to assist a colleague. These actions are not evidenced by title but by indicators such as decreased burnout and increased resiliency. We did not attempt to measure this type of level 4 result but hope to have planted the seeds for this type of leader. The long-term legacy of any leadership program is the impact on organizations and people over time.

“Though I do not believe that a plant will spring up where no seed has been, I have great faith in a seed. Convince me that you have a seed there, and I am prepared to expect wonders.” — Henry David Thoreau

## Supplementary Information

ESM 1 (DOCX 39 kb)

ESM 2 (DOCX 36 kb)

ESM 3 (DOCX 38 kb)

ESM 4 (DOCX 36 kb)

ESM 5 (DOCX 38 kb)

## Declarations

This project was reviewed by the University of Chicago Institutional Review Board and found to be exempt, IRB #16-1299. Date: September 9, 2016.
