# Supplementary material for: The ACLGIM LEAD Program: a Leadership Program for Junior-Mid-Career Faculty
Source: J Gen Intern Med. 2021 Jun 9;36(8):2443–7. doi: 10.1007/s11606-021-06918-y (PMC8342749; doi:10.1007/s11606-021-06918-y)
Supplement: Supplementary file 1 — (DOCX 39 kb) [file 11606_2021_6918_MOESM1_ESM.docx]

**Appendix A: LEAD Workshops* – Year, Topic, and Speaker Institution/Credentials**

| Year | Topic | Speaker Institution/Credentials |
| --- | --- | --- |
| 2014 | Influence | Center for Creative Leadership  San Diego, CA |
|  | Public Narrative | Primary Care Progress  Cambridge, MA |
|  | Collaboration | Univ of California San Diego, Rady School of Management, San Diego, CA |
| 2015 | Succession Planning | Ivey Business School, Western University Ontario, Canada |
|  | Interdisciplinary Teams | Rotman School of Management  University of Toronto |
|  | Leading in Crisis | Brigadier General Officer, United States Air Force (Retired) |
| 2016 | Communication in Leadership | Florida Atlantic University College of Business Boca Raton, FL |
|  | Charisma and Leadership | University of Miami School of Business  Miami, FL |
|  | Personalities in Leadership | Florida International University Center for Leadership, College of Business, Miami, FL |
| 2017 | Self-Presentation in Leadership | Johns Hopkins Carey Business School  Baltimore, MD |
|  | Communication in Leadership | Burness Communications  Bethesda, MD |
|  | Leadership Effectiveness | University of Maryland Center for Leadership Innovation and Change, Smith School of Business, Baltimore, MD |
| 2018 | Motivation and Effectiveness | Center for Character and Leadership Development, USAFA, CO |
|  | Leadership Accountability | Public Affairs, FE Warren AFB  Cheyenne, WY |
|  | Resilience and Emotional Intelligence in Leadership | Collaborative Growth  Denver, CO |
| 2019 | Failure on the Road to Success | Johns Hopkins Carey Business School  Baltimore, MD |
|  | Effective Public Messaging | Burness Communications  Bethesda, MD |
|  | Mindfulness Based Leadership | Georgetown University Institute for Transformational Leadership, Washington D.C. |

*Workshops in 2014-2016 were a 90-minute format. Format for SGIM workshops changed in 2017-2019 to a 60-minute format.
