# Supplementary material for: The ACLGIM LEAD Program: a Leadership Program for Junior-Mid-Career Faculty
Source: J Gen Intern Med. 2021 Jun 9;36(8):2443–7. doi: 10.1007/s11606-021-06918-y (PMC8342749; doi:10.1007/s11606-021-06918-y)
Supplement: Supplementary file 2 — (DOCX 36 kb) [file 11606_2021_6918_MOESM2_ESM.docx]

**Appendix B: Sample of LEAD Scholar Projects**

| Lead a new Primary Care Innovation Lab to achieve tangible outcomes and develop an approach for the team to work toward innovations. |
| --- |
| Develop a robust and concrete mentoring program to improve physician satisfaction and hospitalist retention |
| Undertake a patient centered medical home transformation to include focusing on managing the expectations and need of a diverse team, build a toolkit for decision making, and get buy-in from the team. |
| Expand components of leadership development to the health professions students on campus. |
| Create a visual management and tracking system for the clinic using LEAN principles. |
| Develop a new Observation Unit and measure its quality, service, and financial goals. |
| Practice decision-making capacities that require a spine that will enable making tough calls in positions of greater responsibility. |
| Overhaul of an inefficient and unorganized outpatient clinic. |
| Develop an Education Evaluation Core to provide clinician educators expertise in programmatic and curricular evaluation, enhanced ability to apply for educational grants, and help with publications to assist in promotion. |
| Transitioning a residency practice to the new site. |
| Set up a Women’s Comprehensive Health Center - placing individuals in key roles, developing infrastructure, clinic operations and a strong marketing strategy. |
| Learn how to network, how to mentor others, and how to collaborate with others. |
