# Supplementary material for: The ACLGIM LEAD Program: a Leadership Program for Junior-Mid-Career Faculty
Source: J Gen Intern Med. 2021 Jun 9;36(8):2443–7. doi: 10.1007/s11606-021-06918-y (PMC8342749; doi:10.1007/s11606-021-06918-y)
Supplement: Supplementary file 3 — (DOCX 38 kb) [file 11606_2021_6918_MOESM3_ESM.docx]

**Appendix C: Program Evaluation Survey**

| *Q1. The following components of the LEAD Program positively impacted my personal and leadership development:*  ACLGIM Hess Institute  LEAD Workshops held during Annual Meeting  Asynchronous online curriculum (Harvard Business Review article readings and online discussion)  LEAD Coach  Leadership Interview (interview a leader at your home institution)  Leadership project  Networking within the LEAD Cohort  Other (Please Specify) |
| --- |
| *Q2. Rate your level of agreement with the following statements. (Likert scale 1-4 with 1 strongly disagree, 2 disagree, 3 agree, 4 strongly agree.)*  The LEAD program was helpful to my personal development as a leader.  I gained a better understanding of what it means to be a good leader as a result of the LEAD Program.  I would recommend the LEAD program to junior faculty and mid-career faculty interested in developing their leadership skills |
| **Q3: Rate your level of agreement with the following statements. (Likert scale 1-4 with 1 strongly disagree, 2 disagree, 3 agree, 4 strongly agree.)*  Through participation in the LEAD program, I…  Developed a personal vision or mission.  Have been more effective at leading meetings.  Have been more reflective as a leader.  Ambetter able to create an inclusive and productive working climate.  Have applied the principles of leadership in my work.  Started/strengthened a national network of colleagues with an interest in leadership.  Identified personal strengths, limitations and biases and how these affect my leadership.  Have become a more effective leader. |
| *Q4.* *What improvements (content or delivery) would you like to see with the LEAD program.* (please describe) |
| *Q5. What was your rank when you started LEAD?*  Instructor  Clinical Asst  Clinical Assoc  Clinical Professor  Asst Professor  Tenured Associate  Tenured Professor  Retired  Left Medicine  Other |
| *Q6. What was your current rank?*  Instructor  Clinical Asst  Clinical Assoc  Clinical Professor  Asst Professor  Tenured Associate  Tenured Professor  Retired  Left Medicine  Other |
| *Q7. What leadership role(s) do you hold? (select all that apply, and please indicate which roles are new since your participation in LEAD)*  Clerkship/course director  Division or Section Chief  Department Chair  Residency/fellowship Program Director  Assistant or Associate Program Director  Medical Director of Inpatient Service line/Clinic  Research Center/Quality Center Director  Assistant or Associate Dean  Dean  Hospital/Clinic Administration  N/A  Other |
| *Q8: Which of the following SGIM leadership positions have served in?*  (select all that apply, and please indicate which roles which are new since your participation in LEAD)  SGIM Regional Leadership (President/Treasurer/Secretary/Membership Director)  SGIM Council (President/Treasurer/Secretary/Membership Director, At Large Council Member, etc.)  National SGIM Council Member  Board of Regional Leaders  ACLGIM member  ACLGIM board member  SGIM committee/commission member/leader  SGIM work group member/leader  SGIM interest group leader  LEAD workshop organizer/faculty at SGIM annual meeting  Mentor for CAP or other mentorship programs  Coach for ACLGIM LEAD program  SGIM Forum or ACLGIM Leadership Forum Associate Editor  Other |
| *Q9: What year did you complete the LEAD program?*  2015  2016  2017  2018  2019 |
| *Q10: Which of the following best describes your clinical work? (select one)*  Hospitalist (nearly all inpatient)  Ambulatory care (nearly all outpatient primary care)  Non-primary care – outpatient (urgent care, referral clinic, e.g.)  Both ambulatory and inpatient care  Not applicable  Other (please specify) |
| *Q11: How many years out from residency/fellowship are you?*  0-2  3-5  6-10  10-15  >15 |
| *Q12 Gender*  Male  Female  Non-binary  Prefer not to answer  Pefer to self-describe: |
| *Q13: Race/Ethnicity*  African American/Black  American Indian/Alaskan Native  Asian  Asian Indian  Caucasian/White  Hispanic/Latino/Spanish  Pacific Islander/Native Hawaiian  Prefer Not to Answer  Prefer to self-describe |
| *Q14: Is there any portion of the LEAD program that has been a disappointment for you?* |
| *Q15: Is there any portion of the LEAD program that has been surprising or unexpected?* |
