# Supplementary material for: The ACLGIM LEAD Program: a Leadership Program for Junior-Mid-Career Faculty
Source: J Gen Intern Med. 2021 Jun 9;36(8):2443–7. doi: 10.1007/s11606-021-06918-y (PMC8342749; doi:10.1007/s11606-021-06918-y)
Supplement: Supplementary file 4 — (DOCX 36 kb) [file 11606_2021_6918_MOESM4_ESM.docx]

**Appendix D: Comments from Participants – representative sample**

| ***Participant Comments - Positive*** |
| --- |
| *“I found it most comforting that many parts of leadership can be learned.”*  *“Definitely led to positive changes in my career.”*  *“I started to see the facets of effective and ineffective leaders in my midst through the descriptions in the papers and developed a vision of what an outstanding leader looks like.”*  *“There was a lot of self-reflection to determine one’s own weaknesses, strengths, and what we felt like were important aspects of leadership.”*  *“The diversity of insights and opinions are valuable.”*  *“I have really enjoyed the readings and the interactions with my mentor.”*  *“Great to have this community of peers; I hope that we can remain in touch after the year is over.”*  *“The vast leadership experience and support from the cohort has been wonderful.”*  *“Networking was the most important part of the LEAD program (for me).”* |
| ***Participant Comments - Negative*** |
| *“I would like to network more between the two national meetings.”*  *“Mid-year things got hectic for everyone.”*  *“When people did respond after some silence, they used it as a way to tell the rest of us how they were so busy…That was not very helpful. Everyone is busy.”*  *“After the program, I wasn’t sure how to stay involved.”*  *“I was most disappointed in myself because I wasn't able to keep up.”*  *“My LEAD coach is a wonderful human being but was not routinely available for meetings.”* |
| ***Participant Comments – Suggestions for Improvement*** |
| *“More intense in-person sessions with just LEAD cohort (at national meeting).”*  *“Consider strengthening the alumni programming.”*  *“Perhaps a mini-conference call or video-call (during the year).”*  *“Perceived accountability.”* |
