# Supplementary material for: The ACLGIM LEAD Program: a Leadership Program for Junior-Mid-Career Faculty
Source: J Gen Intern Med. 2021 Jun 9;36(8):2443–7. doi: 10.1007/s11606-021-06918-y (PMC8342749; doi:10.1007/s11606-021-06918-y)
Supplement: Supplementary file 5 — (DOCX 38 kb) [file 11606_2021_6918_MOESM5_ESM.docx]

**Appendix E: LEAD Participant Career Advancement from Surveyed Participants**

|  | BEFORE LEAD | AFTER LEAD |
| --- | --- | --- |
| Academic Rank |  |  |
| Instructor | 5.1% | 2.6% |
| Assistant Professor | 71.8% | 51.3% |
| Associate Professor | 23.1% | 46.1% |
| Professor | 0 | 0 |
| Leadership positions |  |  |
| Division or section chief | 2 | 3 |
| Residency/fellowship program director | 7 | 8 |
| Medical director of inpatient service line/clinic | 10 | 11 |
| Assistant or Associate Dean | 2 | 3 |
| Hospital/Clinic Administration | 5 | 8 |
| Other – Associate Chief of Education, DGIM, Director of Faculty Development, Associate Chief of GIM, Associate Director of Clinical programs, Vice Chief for Education, DGIM, Vice Chair of Clinical Affairs, Internal Medicine, Vice Chair for Leadership Development, Director of strategic Leadership Development of the Enterprise, Thread Director (longitudinal curriculum); Vice chair for clinical affairs | 2 | 7 |
| SGIM Leadership roles |  |  |
| SGIM Regional Leadership | 2 | 4 |
| SGIM National leadership | 1 | 2 |
| ACLGIM Member | 2 | 7 |
| ACLGIM Executive Council Member | 0 | 1 |
| SGIM Committee/Commission member/Leader | 10 | 9 |
| SGIM work group member/leader | 4 | 6 |
| SGIM interest group leader | 6 | 7 |
| LEAD workshop organizer/faculty at SGIM annual meeting | 0 | 3 |
| Mentor for CAP/mentorship program/LEAD program | 1 | 6 |
| SGIM Forum/ACLGIM Leadership Forum Associate Editor | 2 | 4 |
